# Chronic Administration of Oil Palm (*Elaeis guineensis*) Leaves Extract Attenuates Hyperglycaemic-Induced Oxidative Stress and Improves Renal Histopathology and Function in Experimental Diabetes

**DOI:** 10.1155/2012/195367

**Published:** 2012-11-22

**Authors:** Varatharajan Rajavel, Munavvar Zubaid Abdul Sattar, Mahmood Ameen Abdulla, Normadiah M. Kassim, Nor Azizan Abdullah

**Affiliations:** ^1^Department of Pharmacology, Faculty of Medicine, University of Malaya, 50603 Kuala Lumpur, Malaysia; ^2^School of Pharmaceutical Sciences, Universiti Sains Malaysia, 11800 Penang, Malaysia; ^3^Department of Molecular Medicine, Faculty of Medicine, University of Malaya, 50603 Kuala Lumpur, Malaysia; ^4^Department of Anatomy, Faculty of Medicine, University of Malaya, 50603 Kuala Lumpur, Malaysia

## Abstract

Oil palm (*Elaeis guineensis*) leaves extract (OPLE) has antioxidant properties and because oxidative stress contributes to the pathogenesis of diabetic nephropathy (DN), we tested the hypothesis that OPLE prevents diabetes renal oxidative stress, attenuating injury. Sprague-Dawley rats received OPLE (200 and 500 mg kg^−1^) for 4 and 12 weeks after diabetes induction (streptozotocin 60 mg kg^−1^). Blood glucose level, body and kidney weights, urine flow rate (UFR), glomerular filtration rate (GFR), and proteinuria were assessed. Oxidative stress variables such as 8-hydroxy-2′-deoxyguanosine (8-OHdG), glutathione (GSH), and lipid peroxides (LPO) were quantified. Renal morphology was analysed, and plasma transforming growth factor-beta1 (TGF-**β**1) was measured. Diabetic rats demonstrated increase in blood glucose and decreased body and increased kidney weights. Renal dysfunction (proteinuria, elevations in UFR and GFR) was observed in association with increases in LPO, 8-OHdG, and TGF-**β**1 and a decrease in GSH. Histological evaluation of diabetic kidney demonstrated glomerulosclerosis and tubulointerstitial fibrosis. OPLE attenuated renal dysfunction, improved oxidative stress markers, and reduced renal pathology in diabetic animals. These results suggest OPLE improves renal dysfunction and pathology in diabetes by reducing oxidative stress; furthermore, the protective effect of OPLE against renal damage in diabetes depends on the dose of OPLE as well as progression of DN.

## 1. Introduction 

Diabetic nephropathy (DN) is a major microvascular complication of diabetes and is the leading cause of chronic renal failure and end-stage renal disease (ESRD) worldwide. Traditionally, DN has been described as a glomerular disease with the following stages: glomerular hyperfiltration, incipient nephropathy, microalbuminuria, overt proteinuria, and ESRD [1]. The morphological changes that occur in DN include glomerular hypertrophy, thickening of glomerular basement membrane, and mesangial expansion. These morphological changes probably give rise to proteinuria, renal dysfunction, and eventually to development of glomerulosclerosis and tubulointerstitial fibrosis [2]. At present no adequate treatment is available for diabetic renal injury [3–7] and thus other agents that can affect the molecular mechanisms which contribute to the pathogenesis of DN are essential. 

Studies in both humans and animal models clearly implicate the contribution of oxidative stress to the pathogenesis of DN [8–11]. Chronic hyperglycaemia is probably the most important factor in the generation of oxidative stress. Oxidative stress has an important role in the pathogenesis of glomerular and tubular function and structural abnormalities, these alterations include extracellular matrix (ECM) deposition in the mesangium, promotion of a hypoxic environment via microvascular damage, induction of cellular oxidant injury and, ultimately, promotion of tubulointerstitial fibrosis by activation of transforming growth factor *β*1 (TGF-*β*1) [11–15]. In view of the potential hazards of sustained hyperglycaemic-induced oxidative stress to the kidney, a potent novel antioxidant to treat DN would be highly desirable. 

Treatments with antioxidants such as green tea and antioxidant vitamins have however produced marginal benefit in preventing the progression of diabetic renal complications [16] and human studies have shown that high doses of vitamin E failed to impart positive effect [17, 18]. The outcomes of these studies thus lay emphasis on the significance of developing better novel antioxidant treatments for reducing the incidence and attenuating the progression of diabetic complications such as DN.

Oil palm (*Elaeis guineensis*) leaves are abundant, under-utilised, by-products of the palm oil industry in tropical countries including Indonesia, Thailand, Malaysia, Africa, and South America, and the oil palm leaves have been used for decades as ruminant feed without any reports of toxicity. The methanol extract of these leaves is rich in flavonoids and catechins [19]; catechins are polyphenolic compounds which possess antioxidant activity that is several folds higher than that of vitamins C and E. Indeed the antioxidant properties of oil palm leaves extract (OPLE) which is rich in these compounds have been documented [20, 21]. 

The aim of the present study was therefore to investigate the effectiveness of OPLE in attenuating hyperglycaemia-mediated oxidative stress and renal dysfunction in streptozotocin-induced diabetic rats. Impact of this treatment on metabolic parameters and renal function was determined. In addition, morphologic change was observed in renal tissues and oxidative stress markers (8-hydroxy-2′-deoxyguanosine, lipid peroxides, and glutathione) were measured in urine and renal tissues. TGF-*β*1, a key mediator of ECM accumulation in diabetic kidney, was measured in plasma. 

## 2. Materials and Methods

### 2.1. Chemicals

Standardised OPLE was obtained from Nova Laboratories, Malaysia (Batch no: WH 1446). Streptozotocin (STZ) was purchased from Sigma-Aldrich (St. Louis, MO, USA). Enzyme-linked immunosorbent assay for the determination of glutathione (GSH), lipid peroxides (LPO), and 8-hydroxy-2′-deoxyguanosine (8-OHdG) was obtained from Cayman Chemicals (Ann Arbor, MI, USA). Enzyme-linked immunosorbent assay for the determination of transforming growth factor-beta 1 (TGF-*β*1) was obtained from Abnova (Walnut, USA). Other standard chemicals were obtained from common commercial suppliers.

### 2.2. Preparation of OPLE

Ethanolic fraction of oil palm leaves was prepared as described by the manufacturer: briefly 50 g of dried oil palm leaves was macerated with 500 mL of water and ethanol (3 : 7 v/v) at about 60–65°C for approximately 60 min was filtered. The extract was applied to column adsorptive chromatography (Amberlite XAD 16 HP, Rohm & Haas) and eluted with water: isopropanol (3 : 7 v/v) with a constant flow rate of 25 mL min^−1^, at 60°C and a pressure of 0.5–1 bar. The eluant was concentrated in a rotary evaporator and dried in a vacuum oven at 60°C for 8 h to yield a 2.5 g yellow-beige powder. The powder extract was stored in a refrigerator at 2–8°C for use in subsequent experiments. Preliminary screening revealed the presence of 1.1%  (−) catechin gallate and 1.5% ferulic acid in the powder extract.

### 2.3. Experimental Animals and General Preparations

All experimental procedures were approved and complied with the Guidelines for the Care and Use of Laboratory Animals at University of Malaya in Kuala Lumpur (FAR/20101106/NAA-R). Male Sprague-Dawley rats weighing 270–330 g were acclimatized for 1 week before starting any experiment. They were kept under controlled conditions with a 12 h light: dark cycle and at 21–25°C. All rats were allowed unrestricted access to standard rat pellet and tap water. Animals were randomly divided into four groups (*n* = 10 to 15 per group), namely, nondiabetic (N), diabetes control group (D), diabetes group treated with OPLE at a dose of 200 mg kg^−1^ and 500 mg kg^−1^, respectively. A single intraperitoneal (*i.p*.) injection of STZ at a dose of 60 mg kg^−1^ was used to induce diabetes. STZ was dissolved in freshly prepared 0.9% sodium chloride (pH 4.5). Control rats received an equal volume of saline by *i.p.* injection. Diabetes was confirmed after 72 h by measuring blood glucose levels with the use of glucose oxidase reagent strips (one touch glucometer, Accu Chek). Rats with a blood glucose level of >12 mmol L^−1^ were selected for the study. After confirmation of diabetes, the rats were treated with OPLE orally by gavage daily for 4 and 12 weeks. Age-matched nondiabetic and diabetic control groups did not receive any treatment. Blood glucose levels were measured twice in a week. After 4 weeks, the percentages of survivors amongst the diabetic groups were 80%, 80% and 90% in each group, respectively. The percentages of survivors 12 weeks after diabetes induction were 70%, 75%, and 80% in each group, respectively. At the end of the 4 and 12 week periods, the rats were anaesthetised with sodium pentobarbital (60 mg kg^−1^, *i.p.*) for the acute study to examine evidence of renal dysfunction and injury. 

### 2.4. Surgical Preparation and Functional Studies

A tracheostomy was performed to facilitate respiration. The left jugular vein and carotid artery were cannulated for anaesthetic infusion (12.5 mg kg^−1^ h^−1^ at 3 mL h^−1^ in 140 mM NaCl) and arterial pressure recordings (Power Lab Systems and pressure transducer, AD Instruments), respectively while the left femoral artery was cannulated for blood sample collections. The left kidney was exposed via a flank incision and a catheter was placed in the urinary bladder for urine collections. An electromagnetic flow probe was placed on the renal artery for renal blood flow (RBF) measurement (Carolina Square-wave Electromagnetic flowmeter and EP 100 series probe; Carolina Medical, NC, USA). Inulin (10 mg mL^−1^) was included in the infusate. Thereafter, a 2 mL priming dose solution (10 mg mL^−1^ inulin in 140 mM NaCl) was administered to the rat via the jugular vein. Following a 1 h equilibration period, four 30 min clearances were taken. Arterial blood samples were taken at the beginning and at the end of every two clearances. The blood samples were centrifuged and plasma was frozen at −20°C for subsequent analysis. At the end of the study, the rat was killed by a rapid intravenous injection of 1 mL sodium pentobarbital and the right kidney was harvested, dissected into cortex and medulla, and snap frozen in liquid nitrogen for measurement of GSH and LPO. The left kidney was perfused first with ice-cold phosphate buffered saline until cleared of blood and then fixed with 10% formalin for histological evaluation.

### 2.5. Biochemical Analysis

Urinary protein concentrations were determined by biuret reagent, according to the method developed by Doumas et al. [22]. Urine and plasma electrolytes (sodium and potassium) concentrations were analysed using a flame photometer (Sherwood, Model 420, UK) and urine volume was measured gravimetrically. Inulin content in urine and plasma samples was determined according to the method of Somogyi [23] after the samples were deproteinized according to the method proposed by Bojesen [24]. Glomerular filtration rate (GFR) was equated with renal clearance of inulin and was expressed as mL min^−1^ g^−1^ kidney weight.

### 2.6. Reduced GSH in Kidney

Cytosolic reduced GSH in renal cortex homogenates was measured using a glutathione assay kit from Cayman Chemicals according to the manufacturer's protocol. The sample (100–150 *μ*g) is deproteinized using metaphosphoric acid, and the amount of the yellow coloured 5-thio-2-nitrobenzoic acid produced in the supernatant was measured at 410 nm. 

### 2.7. LPO in Kidney

LPO in the renal cortex was measured colourimetrically using a Cayman's assay kit. Briefly, renal cortex was homogenized in HPLC-grade water and LPO was extracted from the homogenates according to the manufacturer's protocol. LPO was measured directly by redox reactions with ferrous ions using the kit, and the resulting ferric ions were detected using thiocyanate ion as the chromogen. 

### 2.8. Urinary Excretion of 8-OHdG

8-OHdG is one of the most common markers for oxidative DNA damage and oxidative stress *in vivo*. ELISA kit from Cayman Chemicals was used to measure 8-OHdG levels in 2 h urine samples. The 8-OHdG standards (0.5–80 ng mL^−1^) or 35–50 *μ*L of urine were allowed to incubate for 1 h with monoclonal antibody against 8-OHdG in a microtiter plate precoated with 8-OHdG. After washing the antibodies bound to 8-OHdG in the sample, enzyme-labelled secondary antibody was added to each well and incubated for 1 h followed by washing. The colour that developed by the addition of 3,3′,5,5′-tetramethylbenzidine was measured by absorbance at 450 nm. Urinary 8-OHdG was expressed as total amount excreted in 2 h.

### 2.9. Plasma TGF-*β*1

Plasma samples were collected from the experimental animals on the 4th and 12th weeks. Samples were frozen at −20°C until analysis. ELISA kit was used to measure plasma TGF-*β*1 concentration according to manufacturer's protocol. Each 50 *μ*L of plasma sample was first acid activated by incubation with 1 *μ*L of 1.0 N HCl for 15 min at room temperature and neutralized by 1 *μ*L of 1 N NaOH for the activation of the plasma TGF-*β*1 to the immunoreactive form. Plasma TGF-*β*1 is expressed as total amount excreted in pg mL^−1^.

### 2.10. Morphologic Study

After formalin fixation, renal tissues were embedded in paraffin. Tissue sections were cut at 5 *μ*m thickness using a microtome, dewaxed, and stained with periodic acid-Schiff (PAS) and Masson's trichrome. Renal morphology changes within the glomeruli and interstitial areas were assessed with the aid of a Nikon Eclipse 80i light microscope, using a semiquantitative scoring method [25, 26].

### 2.11. Statistical Analysis

Data are shown as mean ± SEM. The mean values were compared among the 4 groups using one way analysis of variance (ANOVA), followed by Tukey Multiple Comparison Test (Graph Pad Prism). Experimental differences were considered statistically significantif *P* < 0.05. 

## 3. Results

### 3.1. Metabolic Parameters

Table 1 shows the effects on the changes in body and kidney weights of diabetic rats after oral administration of 200 mg kg^−1^ and 500 mg kg^−1^ OPLE. The mean body weight in diabetic rats was 1.2-fold and 1.8-fold lower than in nondiabetic rats at 4 and 12 weeks after induction of diabetes, respectively; however, the difference was only significant at 12 weeks (*P* < 0.001). In contrast, kidney weight normalised by 100 g body weight in diabetic group was significantly heavier than in nondiabetic rats by 1.4-fold (*P* < 0.01) and 2.2-fold (*P* < 0.001) at 4 and 12 weeks of diabetes, respectively. OPLE did not affect the mean body weight or kidney to body weight ratio in diabetic rats at the doses tested for both the 4-week and 12-week experimental duration. Even though the difference is not statistically significant, OPLE at 500 mg kg^−1^showed a slight tendency to reduce renal enlargement in rats with 12 weeks diabetes.

STZ-induced diabetes demonstrated a tremendous elevation of blood glucose level in all the diabetic groups, random glucose levels in the whole blood of diabetic rats, and diabetic rats treated with OPLE at 200 mg kg^−1^ and 500 mg kg^−1^ were significantly higher than that of nondiabetic rats (6.5-, 6.8- and 6.6-fold, respectively, all *P* < 0.001) at 4 weeks after induction of diabetes. Similarly, 12 weeks after the induction of diabetes, random blood glucose level was significantly higher in all the diabetic groups compared to that of nondiabetic group (all *P* < 0.001). Treatment of diabetic animals with OPLE at the doses tested for 4 and 12 weeks did not affect random blood glucose level (Table 1). Mean arterial pressure was comparable between the four study groups (Table 1) at 4 and 12 weeks.

### 3.2. Renal Haemodynamic

Renal blood flow (RBF) of various groups in the present study is listed in Table 2. RBF did not differ among the four study groups at 4 weeks. Conversely, RBF was significantly increased in diabetic rats compared with nondiabetic rats at 12 weeks (1.5-fold, *P* < 0.001). The increase in RBF was normalised by treatment with 200 mg^−1 ^kg^−1^ and 500 mg^−1 ^kg^−1^ OPLE (*P* < 0.01 and *P* < 0.001, resp.). 

It is well established that early stages of DN are associated with increases in glomerular filtration rate (GFR), both clinically and experimentally. Rats with 4-week diabetes in the present study exhibited elevated values for GFR compared with those found in nondiabetic rats (1.6-fold, *P* < 0.01), as evaluated by inulin clearance (Table 2). Hyperfiltration was prevented by treatment with OPLE at 200 mg kg^−1^ and 500 mg kg^−1^ (both *P* < 0.05). These results indicate that the rise in RBF may be responsible for the hyperfiltration in diabetic rats, although a rise in glomerular capillary pressure might also contribute.

Likewise, GFR was significantly greater (1.7-fold, *P* < 0.01) in rats of 12-week diabetes than in nondiabetic rats but only the higher dose of OPLE (500 mg kg^−1^) suppressed the increase in GFR in diabetic rats (*P* < 0.05). 

### 3.3. Renal Excretory Function

Untreated diabetic rats were polyuric (Table 2); urine flow rate (UFR) increased remarkably in diabetic rats when compared to that of nondiabetic rats at both 4 weeks (2.8-fold, *P* < 0.001) and 12 weeks (2.7-fold, *P* < 0.001). This event may be the outcome of hyperfiltration. Treatment with OPLE at both the concentrations tested almost prevented the rise in urine flow rate at 4 weeks (both *P* < 0.01) but only the higher dose of OPLE ameliorated the elevation in urine flow rate in diabetic rats at 12 weeks (*P* < 0.05). 

To substantiate the beneficial effect of OPLE treatment on renal dysfunction in diabetes, we measured urinary protein excretion in the four study groups (Table 2). Urinary protein excretion increased significantly in diabetic rats compared with nondiabetic rats at both 4 weeks (5.1-fold, *P* < 0.001) and 12 weeks (4.1-fold, *P* < 0.001), indicating a modification in glomerular barrier characteristics. Proteinuria induced by diabetes was significantly reduced by treatment with OPLE at both concentrations tested at 4 weeks (both *P* < 0.001); furthermore, the higher dose reduced the excretion to almost nondiabetic value. At 12 weeks, OPLE at both concentrations tested ameliorated the increase in urinary protein excretion in diabetic rats (*P* < 0.05 for 200 mg kg^−1^ and *P* < 0.01 for 500 mg kg^−1^). This finding shows that treatment with OPLE prevents the development of proteinuria, a hallmark of DN, in early diabetes.

We evaluated fractional sodium excretion to obtain some information on the integrity of tubular reabsorptive function. Our results showed fractional sodium excretion did not differ among the four experimental groups at both 4 and 12 weeks (Table 2).

### 3.4. Parameters of Oxidative Stress

In this study, we did not quantify reactive oxygen species (ROS) directly but alternatively measured the formation of oxidative damage products or endogenous end-products of ROS to address the effect of OPLE on STZ-diabetes induced oxidative stress.

#### 3.4.1. 8-Hydroxy-2-deoxyguanosine (8-OHdG)

**Figure 1:**
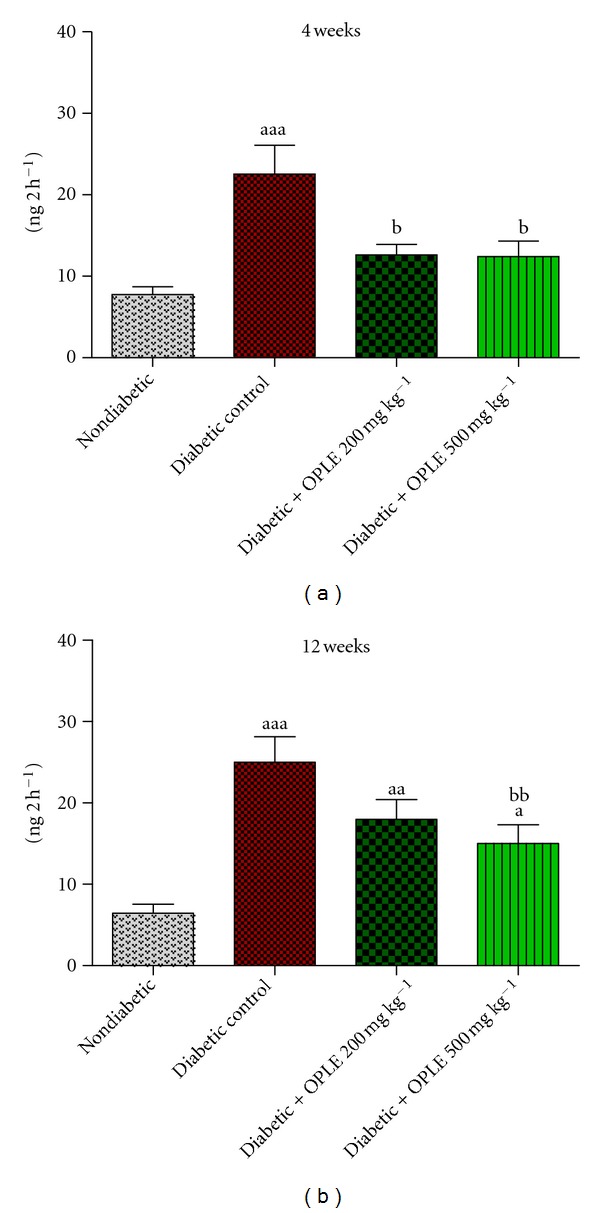
Effect of OPLE on urinary 8-OHdG concentration. Data are expressed as mean ± SEM (*n* = 6 per group). ^a^ *P* < 0.05; ^aa^ *P* < 0.01; ^aaa^ *P* < 0.001 versus corresponding nondiabetic; ^b^ *P* < 0.05; ^bb^ *P* < 0.01 versus corresponding diabetes control.

We examined the oxidative DNA damage in kidney of STZ-induced diabetic rats by measuring the levels of 8-OHdG in urine samples. The levels of urinary 8-OHdG (Figure 1) were significantly greater in diabetic rats than in nondiabetic rats at both 4 weeks (22.56 ± 3.51 ng 2 h^−1^ versus 7.77 ± 0.93 ng 2 h^−1^, *P* < 0.001) and 12 weeks after the onset of diabetes (25.01 ± 3.13 ng 2 h^−1^ versus 6.45 ± 1.09 ng 2 h^−1^, *P* < 0.001). These levels were reduced significantly in diabetic rats treated with 200 mg kg^−1^ and 500 mg kg^−1^ OPLE for 4 weeks to amounts that were not significantly different from that of nondiabetic rats (12.62 ± 1.31 ng 2 h^−1^ and 12.40 ± 1.93 ng 2 h^−1^, resp.). In contrast, only the higher dose of OPLE (500 mg kg^−1^) reduced the 8-OHdG level significantly in 12-week diabetic rats compared to untreated diabetic rats (15.02 ± 2.31 ng 2 h^−1^, *P* < 0.01). These findings indicate that antioxidant treatment with OPLE ameliorates the oxidative stress-induced DNA damage in the kidney in the early stage of experimental diabetes.

#### 3.4.2. Lipid Peroxides (LPO)

**Figure 2:**
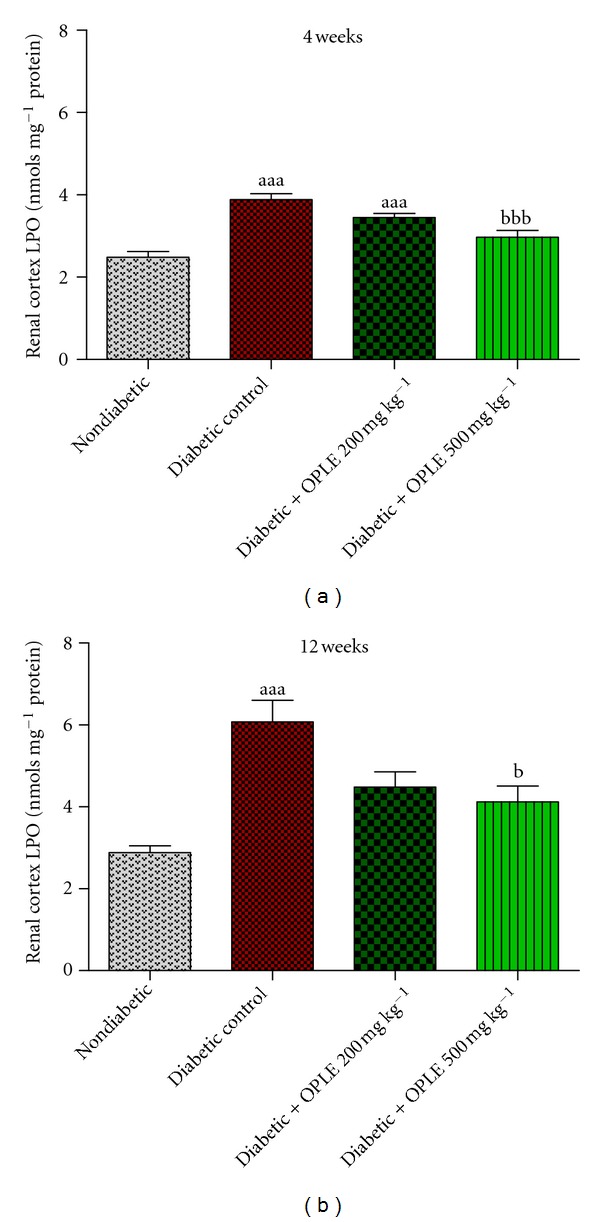
Effect of OPLE on renal cortical LPO concentration. Data are expressed as mean ± SEM (*n* = 6 per group). ^aaa^ *P* < 0.001 versus corresponding nondiabetic; ^b^ *P* < 0.05, ^bbb^ *P* < 0.001 versus corresponding diabetes control.

Renal cortical LPO were significantly higher in diabetic rats than in nondiabetic rats (Figure 2) at both 4 weeks (3.88 ± 0.15 nmols mg^−1^ protein versus 2.48 ± 0.14 nmols mg^−1^ protein, *P* < 0.001) and 12 weeks (6.08 ± 0.53 nmols mg^−1^ protein versus 2.88 ± 0.17 nmols mg^−1^ protein, *P* < 0.001) after induction of diabetes. While 200 mg kg^−1^ OPLE did not affect renal cortical LPO levels in 4-week and 12-week diabetic rats, 500 mg kg^−1^ reduced significantly renal cortical LPO levels in diabetic rats at both 4 weeks (2.97 ± 0.17 nmols mg^−1^ protein, *P* < 0.001) and 12 weeks (4.12 ± 0.39 nmols mg^−1^ protein, *P* < 0.05).

#### 3.4.3. Glutathione (GSH)

**Figure 3:**
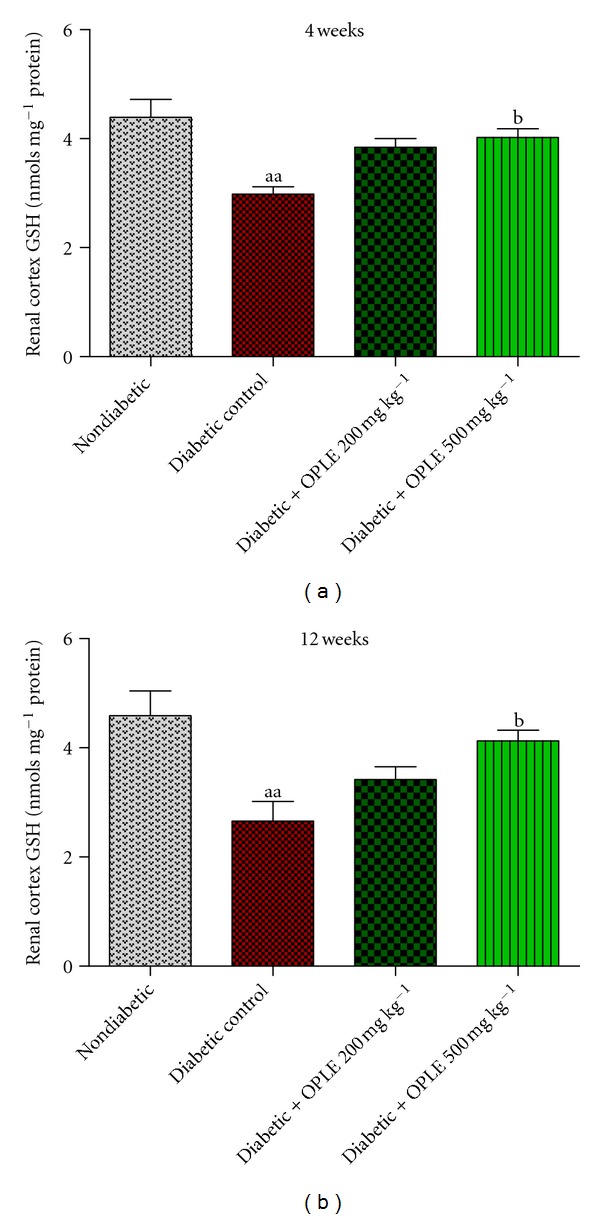
Effect of OPLE on kidney (renal cortex) GSH concentration. Data are expressed as mean ± SEM (*n* = 6 per group). ^aa^ *P* < 0.01 versus corresponding nondiabetic; ^b^ *P* < 0.05 versus corresponding diabetes control.

GSH constitute part of the endogenous antioxidant defence system, the GSH redox cycle plays a major role in scavenging hydrogen peroxide (H_2_O_2_) under physiological conditions. To establish the effect of OPLE on endogenous antioxidant defence system in diabetes, we measured renal GSH content. As demonstrated in (Figure 3), STZ-induced diabetes reduction in renal cortical GSH content was improved by 500 mg kg^−1^ OPLE and not by 200 mg kg^−1^ OPLE in diabetic rats at both 4 weeks (4.02 ± 0.16 nmols mg^−1^ protein versus 2.98 ± 0.13 nmols mg^−1^ protein in untreated diabetic rats, *P* < 0.05) and 12 weeks (4.13 ± 0.19 nmols mg^−1^ protein versus 2.65 ± 0.36 nmols mg^−1^ protein in untreated diabetic rats, *P* < 0.05). 

### 3.5. Transforming Growth Factor Beta-1 (TGF-*β*1)

**Figure 4:**
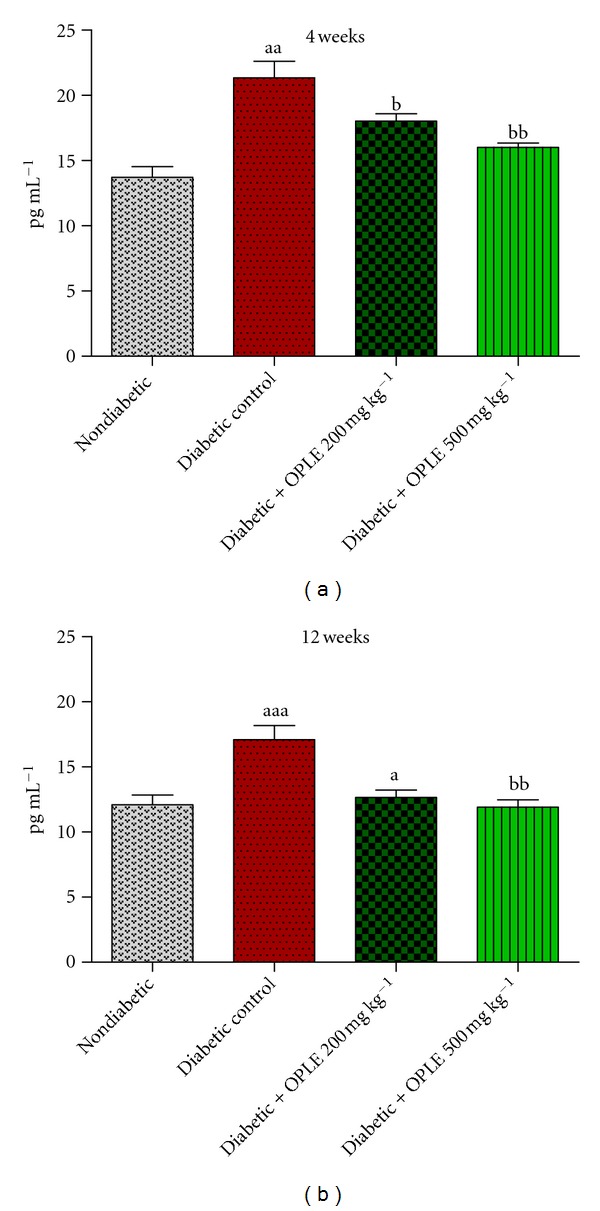
Plasma TGF-*β*1 concentration. Data are expressed as mean ± SEM (*n* = 6 per group). ^a^ *P* < 0.05; ^aa^ *P* < 0.01; ^aaa^ *P* < 0.001 versus corresponding nondiabetic; ^b^ *P* < 0.05; ^bb^ *P* < 0.01 versus corresponding diabetes control.

TGF-*β*1, a fibrogenic cytokine, is purported to be a major mediator of the hypertrophic changes in diabetic kidney disease. Thus in accord with renal dysfunction and the increased markers of oxidative stress (urinary 8-OHdG excretion and renal cortical LPO), significant higher concentrations of TGF-*β*1 were detected in plasma of diabetic rats in our study than in nondiabetic rats (Figure 4) at both 4 weeks (17.09 ± 1.10 pg mL^−1^ versus 12.09 ± 0.75 pg mL^−1^, *P* < 0.01) and 12 weeks (21.34 ± 1.27 pg mL^−1^ versus 13.71 ± 0.81 pg mL^−1^, *P* < 0.001). Treatment of diabetic rats with OPLE at both concentrations for 4 weeks normalised plasma TGF-*β*1 concentrations (12.65 ± 1.12 pg mL^−1^ and 11.90 ± 0.56 pg mL^−1^ with 200 mg kg^−1^ OPLE and 500 mg kg^−1^ OPLE, respectively; *P* < 0.05 and *P* < 0.01, resp.). OPLE at 200 mg kg^−1^ did not have significant effect on plasma TGF-*β*1 concentrations in diabetic rats at 12 weeks but OPLE at 500 mg kg^−1^ normalised the plasma TGF-*β*1 concentrations (16.01 ± 0.33 pg mL^−1^, *P* < 0.001).

### 3.6. Renal Histology

**Figure 5:**
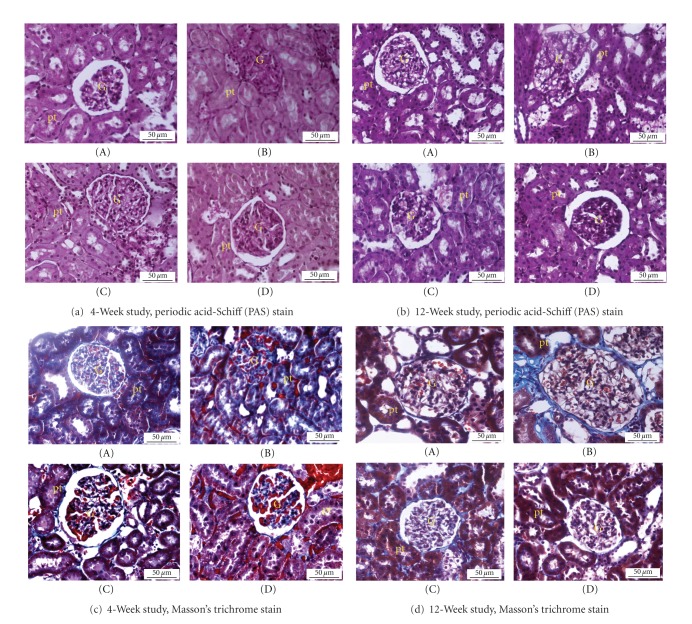
Photomicrographs of kidneys stained with periodic acid-Schiff (PAS) and Masson's trichrome. Nondiabetic (A); diabetic control (B); diabetic + OPLE 200 mg kg^−1^(C); diabetic + OPLE 500 mg kg^−1^(D). Bar = 50 *μ*m.

Figure 5 shows histological observations of kidney sections stained with PAS and Masson's trichrome. PAS-stained sections of the renal cortex 12 weeks after diabetes induction (Figure 5(b)) exhibited marked glomerulosclerosis, characterized by glomerular basement membrane thickening and mesangial expansion with glomerular hypertrophy, compared with nondiabetic controls. Remarkably, treatment with OPLE at both doses (200 mg kg^−1^ and 500 mg kg^−1^) reduced glomerulosclerosis and attenuated the mesangial matrix accumulation in the diabetic group. Morphometric analysis revealed a significant decrease in the mesangial area in OPLE-treated rats and the data clearly demonstrates that OPLE treatment alleviated the mesangial expansion (*P* < 0.05, Table 3). Masson's trichrome-stained section of diabetic kidney on week 4 exhibited increased collagen deposition, tubular dilation, and degeneration of cortical tubules (Figure 5(c)) whilst these changes were not apparent in the nondiabetic kidney. In diabetic rats treated with OPLE, there was very little collagen within the interstitium and no apparent tubular pathology. Marked tubulointerstitial fibrosis characterized by accumulation of extracellular matrix (ECM) protein in cortex and medulla was observed in diabetic kidney on week 12 (Figure 5(d)). There was capillary occlusion, increased proliferation of interstitial fibroblasts, tubular dilatation, and atrophy whereas no apparent changes were detected in kidney of nondiabetic control. Treatment with OPLE reduced tubulointerstitial fibrosis in the renal cortex in the diabetic group when compared to diabetic control (*P* < 0.05, Table 3). 

## 4. Discussion

It is well established that chronic hyperglycaemia is the main determinant in the development and progression of DN, and enhanced oxidative stress has been considered to contribute to the pathological processes of diabetic renal complication [11]. In the diabetic kidney, ROS is generated by several pathways that include glycolysis, polyol pathway flux, uncoupling of nitric oxide synthase (NOS), xanthine oxidase, NAD(P)H oxidase, and advanced glycation. Clinical studies have demonstrated that strict control of hyperglycaemia can reduce the occurrence or progression of DN; however, this is extremely difficult to maintain [2–5]. Therefore, the use of alternate therapies that specifically target oxidative stress implicated in diabetic microvascular complication may be advantageous in addition to a strict glucose control. 

The present study investigated the renoprotective effect of OPLE in experimental diabetes. OPLE is rich in catechins [27, 28], and these polyphenolic compounds are considered to have antioxidant capacity that is several folds higher than that of vitamins C and E [28]. The results of this study provide evidence that OPLE treatment introduced 72 h after diabetes induction and maintained for 4 and 12 weeks, prevented the diabetes-induced renal dysfunction as well as kidney structural injury in diabetic rat. We hypothesised that OPLE may retard renal dysfunction and renal pathology associated with early DN in the STZ-induced diabetic rat, in part, through attenuation of oxidative stress in the kidney, in view of the fact that OPLE suppressed the elevation of oxidative stress markers (8-OHdG, LPO), and improved antioxidant defences as evidenced by increased levels of GSH. In contrast to earlier study [21], these beneficial effects of OPLE could not be ascribed to improvement of the diabetic condition because blood glucose levels were unaffected. 

We tested two different doses of OPLE (200 mg kg^−1^ and 500 mg kg^−1^) to study the effectiveness of this extract in abrogating DN in short-term (4 weeks) and relatively longer-term (12 weeks) diabetes. Both the doses of OPLE produced optimal effects on oxidative stress marker (8-OHdG) and renal dysfunction in short-term diabetes; however, it appeared that the lower dose in contrast to the higher dose was relatively less effective in the longer-term diabetes. This is conceivable given that with progression of the disease, the extent of oxidative stress and its attendant renal dysfunction would be greater and hence would require a higher dose of the extract to counteract. 

The STZ-diabetic rats in our study displayed increased renal perfusion and hyperfiltration which are features of early diabetes in both humans and animal models of diabetes. On the single-nephron level, diabetic hyperfiltration is the result of renal vasodilation predominantly of the preglomerular or afferent resistance vessels [29]. This haemodynamic abnormality was suggested to be mediated by an increase in nitric oxide (NO) due to increased glomerular expression of endothelial constitutive nitric oxide synthase [30, 31]. On the contrary, altered arachidonic acid metabolism has been linked to diabetic hyperfiltration; it was proven that cyclooxygenase (COX)-2 expression was increased in STZ-diabetic rats and inhibition of this enzyme reversed the hyperfiltration in diabetic rats without altering GFR in normal rats [32]. Later studies implicated oxidant peroxynitrite (ONOO^−^) as a stimulus for upregulation of COX-2 in diabetes [33, 34]. Consistent with earlier interventional studies that used antioxidant vitamins in STZ-diabetic rat [35, 36]; the current results demonstrated that hyperfiltration was blunted by OPLE to control values. The mechanism by which OPLE improved hyperfiltration however remains to be researched. A natural antioxidant was reported to reduce 3-nitrotyrosine proteins, a marker of ONOO^−^ production, in renal tissue of STZ-diabetic rat [37]. While this is conjectural, we proposed that OPLE ameliorated hyperfiltration probably due to its antioxidant property that limits the generation or activity of ONOO^−^ and in consequence prevents upregulation of COX-2 and reduces formation of vasodilatory prostanoids. 

Proteinuria, a marker of DN is an important risk factor for progressive renal impairment. We therefore assessed the effect of OPLE on urinary protein excretion to further evaluate the renal protective effect of this extract. Hyperfiltration and alteration in glomerular filtration barrier (alterations in endothelium with its glycocalyx, glomerular basement membrane, and podocyte function) contribute to proteinuria in diabetes mellitus. ROS is believed to be one of the key players involved in the pathogenic pathways that lead to glomerular filtration barrier damage. Glomerular ROS production was increased in experimental diabetes [38] and podocytes produced ROS in response to high glucose [39]. ROS has been shown to directly decrease heparan sulphate proteoglycans production within the glycocalyx [40], disrupt the endothelial glycocalyx [41] and was implicated in early podocyte damage and apoptosis [39]. On the contrary, transgenic overexpression of superoxide dismutase attenuated renal injury, including increases in albumin excretion rate [42] and antioxidant therapies potentially prevented podocyte damage in early DN [43, 44]. In our present study, diabetic rats developed proteinuria by 4 weeks, and OPLE had a positive effect on this parameter. These results may indicate that OPLE can attenuate renal damage in diabetic rats. Moreover, known antioxidants such as polyphenols and catechins are biological active components of OPLE, and these compounds were found to improve proteinuria in diabetes-induced oxidative stress [21, 45].

STZ-diabetic rat manifested renal hypertrophy in the present study and OPLE reduced the renal enlargement although this effect was not significant. Experimental studies suggested that amelioration of oxidative stress by catechin and other antioxidants abrogated renal enlargement in DN [42, 46, 47]. Pathologic hallmarks of early DN include increased glomerular basement membrane thickness and mesangial expansion, and these features are in concordant with our present results. In conjunction with these ultrastructural changes, our present study demonstrated elevation in plasma TGF-*β*1 levels in STZ-diabetic rats. Expansion of the mesangial matrix and thickening of the glomerular basement membrane in DN as a result of excessive deposition of ECM proteins may be due to upregulation of TGF-*β*1 and other growth factors [48]. It is well established that hyperglycaemia-induced ROS activate signal transduction mechanisms and transcription factors and upregulate TGF-*β*1 and ECM genes and proteins [15]. Treatment of diabetic rats with OPLE preserved renal architecture and this was conceivably reflected in suppression of the increases in plasma TGF-*β*1 levels. In corroboration with our findings, antioxidants significantly inhibit high glucose- and H_2_O_2_-induced TGF-*β*1 and fibronectin (glomerular matrix protein) upregulation [49–53].

Quantification of ROS would provide direct evidence of the involvement of oxidative stress in diabetic kidney [46, 54, 55]. Although we did not measure ROS directly, several line of evidence suggested that lipid peroxidation and formation of 8-OHdG constituted a condition of increased oxidative stress [56–58]. Our present findings of increased LPO, 8-OHdG in parallel with compromised concentration of the nonenzymatic antioxidant GSH in diabetic kidney clearly suggested that enhanced oxidative stress was present at an early stage of diabetes. The suggestion that renal oxidative stress in STZ-diabetic rats was a consequence of hyperglycaemia rather than well-established prooxidant effect of STZ itself was supported by findings which demonstrated correction of impaired antioxidative defense and DNA oxidative damage in the diabetic kidney after insulin administration to animals with established STZ-induced diabetes [59, 60]. The diabetes-induced changes in oxidative markers (LPO, 8-OHdG, GSH) in our present study were partially or completely prevented by OPLE, and this is quite consistent with the effect of this compound in other tissues of diabetic animals [21] as well as in other nondiabetic models of oxidative stress [27, 28]. These results indicate that increased oxidative stress which was present in diabetic kidney may be counteracted by OPLE. 

This study indicates that catechins-rich OPLE can modulate oxidative stress caused by hyperglycaemic-induced generation of free radicals in diabetic kidney as well as preventing renal dysfunction and structural injury. DN is a major cause of end-stage renal disease, and it is initially characterised by glomerular hemodynamic abnormalities that result in glomerular hyperfiltration, leading to glomerular damage as evidenced by microalbuminuria. The main advantage of OPLE was that it could ameliorate both hyperfiltration and proteinuria unlike other antioxidant vitamins. Furthermore, renal protection by OPLE in DN depends on the dose of the extract relative to body weight as well as progression of the disease implying that the dose given should correspond to the degree of *in vivo* oxidative stress within the kidney.

## Figures and Tables

**Table 1:** Effects of OPLE on body and kidney weights, random blood glucose level, and mean arterial pressure.

	Nondiabetic	Diabetic control	Diabetic + OPLE	Diabetic + OPLE
			200 mg kg^−1^	500 mg kg^−1^
	(4 week)	(4 week)	(4 week)	(4 week)
	(12 week)	(12 week)	(12 week)	(12 week)
Body weight g	365.0 ± 14.3	309.2 ± 25.4	303.3 ± 37.6	283.3 ± 17.2
	428.3 ± 13.0	235.0 ± 14.6^aaa^	251.7 ± 17.2^aaa^	289.2 ± 14.9^aaa^
Kidney weight g 100 g^−1^ body weight	0.40 ± 0.01	0.57 ± 0.03^aa^	0.53 ± 0.04^a^	0.59 ± 0.04^aa^
	0.32 ± 0.02	0.69 ± 0.02^aaa^	0.62 ± 0.04^aaa^	0.59 ± 0.02^aaa^
Glucose level mmol L^−1^	4.7 ± 0.1	30.7 ± 1.7^aaa^	32.2 ± 0.5^aaa^	31.2 ± 2.1^aaa^
	6.3 ± 0.2	31.0 ± 1.2^aaa^	31.6 ± 1.1^aaa^	33.1 ± 0.2^aaa^
Mean arterial pressure mm Hg	113.2 ± 2.1	115.3 ± 3.2	109.3 ± 3.7	116.7 ± 1.9
	111.5 ± 2.9	116.7 ± 3.3	109.0 ± 5.3	112.2 ± 3.9

Data are expressed as mean ± SEM of six experiments for each group. ^a^ *P* < 0.05; ^aa^ *P* < 0.01; ^aaa^ *P* < 0.001 versus corresponding nondiabetic.

**Table 2:** Effect of OPLE on renal functional parameters.

	Nondiabetic	Diabetic control	Diabetic + OPLE	Diabetic + OPLE
			200 mg kg^−1^	500 mg kg^−1^
	(4 week)	(4 week)	(4 week)	(4 week)
	(12 week)	(12 week)	(12 week)	(12 week)
Renal blood flow mL min^−1^ g^−1^ kidney	1.43 ± 0.10	2.27 ± 0.39	1.72 ± 0.26	1.80 ± 0.03
	1.66 ± 0.14	2.49 ± 0.16^aaa^	1.78 ± 0.06^bb^	1.47 ± 0.05^bbb^
Glomerular filtration rate mL min^−1^ g^−1^ kidney	0.61 ± 0.06	0.96 ± 0.04^aa^	0.67 ± 0.07^b^	0.63 ± 0.09^b^
	0.50 ± 0.05	0.84 ± 0.06^aa^	0.75 ± 0.06^a^	0.58 ± 0.02^b^
Urine flow rate *μ*L min^−1^ g^−1^ kidney	3.75 ± 0.59	10.68 ± 1.30^aaa^	5.27 ± 0.45^bb^	5.80 ± 0.80^bb^
	2.89 ± 0.34	7.84 ± 0.70^aaa^	6.42 ± 0.50^aa^	5.29 ± 0.70^a,b^
Urinary protein excretion mg 2 h^−1^	1.33 ± 0.03	6.83 ± 0.30^aaa^	2.87 ± 0.17^a,bbb^	1.89 ± 0.34^bbb^
	1.13 ± 0.05	4.66 ± 0.73^aaa^	2.92 ± 0 .26^a,b^	2.68 ± 0.21^a,bb^
Fractional sodium excretion %	0.63 ± 0.19	1.34 ± 0.34	0.96 ± 0.22	0.90 ± 0.17
	0.56 ± 0.08	1.08 ± 0.23	0.76 ± 0.24	0.79 ± 0.23

Data are expressed as mean ± SEM of six experiments for each group. ^a^ *P* < 0.05; ^aa^ *P* < 0.01; ^aaa^ *P* < 0.001 versus corresponding nondiabetic; ^b^ *P* < 0.05; ^bb^ *P* < 0.01; ^bbb^ *P* < 0.001 versus corresponding diabetic control.

**Table 3:** Effects of OPLE on renal structure.

	Nondiabetic	Diabetic control	Diabetic + OPLE	Diabetic + OPLE
			200 mg kg^−1^	500 mg kg^−1^
	(4 week)	(4 week)	(4 week)	(4 week)
	(12 week)	(12 week)	(12 week)	(12 week)
Glomerulosclerotic index	0.23 ± 0.03	1.14 ± 0.06^aa^	0.89 ± 0.07^aa^	0.79 ± 0.5^aa,b^
	0.34 ± 0.03	1.36 ± 0.04^aa^	0.93 ± 0.05^aa,b^	0.88 ± 0.04^aa,b^
Tubulointerstitial fibrosis index	0.49 ± 0.05	2.48 ± 0.18^aa^	1.48 ± 0.06^a,b^	1.16 ± 0.03^b^
	0.61 ± 0.06	2.75 ± 0.16^a^	1.31 ± 0.23^b^	1.05 ± 0.14^b^

Data are expressed as mean ± SEM of six experiments for each group. ^a^ *P* < 0.05,^aa^ *P* < 0.01 versus corresponding nondiabetic; ^b^ *P* < 0.05 versus corresponding diabetic control.
